# Neurobiological correlates of schizophrenia-specific and highly pleiotropic genetic risk scores for neuropsychiatric disorders

**DOI:** 10.1038/s41398-025-03440-1

**Published:** 2025-07-05

**Authors:** Lydia M. Federmann, Lisa Sindermann, Sabrina Primus, Federico Raimondo, Konrad Oexle, Janik Goltermann, Juliane Winkelmann, Markus M. Nöthen, Katrin Amunts, Thomas W. Mühleisen, Sven Cichon, Simon B. Eickhoff, Felix Hoffstaedter, Udo Dannlowski, Kaustubh R. Patil, Andreas J. Forstner

**Affiliations:** 1 Institute of Neuroscience and Medicine, Research Centre Jülich, Jülich, Germany; 2 Institute of Human Genetics, University of Bonn, School of Medicine & University Hospital Bonn, Bonn, Germany; 3 Department of Psychiatry and Psychotherapy, University Hospital Bonn, Bonn, Germany; 4 Institute of Neurogenomics, Helmholtz Zentrum München, German Research Center for Environmental Health, Neuherberg, Germany; 5 Neurogenetic Systems Analysis Group, Institute of Neurogenomics, Helmholtz Zentrum München, German Research Center for Environmental Health, Neuherberg, Germany; 6 Institute of Human Genetics, TUM School of Medicine and Health, Technical University of Munich, Munich, Germany; 7 Institute of Systems Neuroscience, Medical Faculty, Heinrich Heine University Düsseldorf, Düsseldorf, Germany; 8 Institute for Translational Psychiatry, University of Münster, Münster, Germany; 9 Munich Cluster for Systems Neurology (SyNergy), Munich, Germany; 10 German Center for Mental Health (DZPG), partner site Munich–Augsburg, Munich–Augsburg, Germany; 11 Cécile and Oskar Vogt Institute for Brain Research, Medical Faculty & University Hospital Düsseldorf, Heinrich Heine University Düsseldorf, Düsseldorf, Germany; 12 Department of Biomedicine, University of Basel, Basel, Switzerland; 13 Institute of Medical Genetics and Pathology, University Hospital Basel, Basel, Switzerland

**Keywords:** Neuroscience, Psychiatric disorders, Genetics

## Abstract

Neuropsychiatric disorders show shared and distinct neurobiological correlates. A cross-disorder genome-wide association study (GWAS) identified 23 highly pleiotropic single-nucleotide polymorphisms (SNPs) that were associated with at least four neuropsychiatric disorders, and 22 SNPs that were associated predominantly with schizophrenia. Exploring their link to brain-related traits might advance understanding their distinct neurobiological processes. Using the UK Biobank data (*n* = 28,952), this study examined the association of both a genetic risk score (GRS) for highly pleiotropic SNPs (PleioPsych-GRS), and a GRS for predominantly schizophrenia-associated SNPs (SCZ-GRS) with 154 measures of subcortical volume, cortical thickness, and surface area as well as 12 outcomes related to mental health. To generate further insights at the individual SNP level, the association between SNPs and brain structure was examined using GWAS summary statistics. The PleioPsych-GRS showed no significant association with brain structure after correction for multiple testing. The SCZ-GRS showed a significant association with an increased surface area of the lateral orbitofrontal region, and an increased volume of the putamen, among others. The PleioPsych-GRS and the SCZ-GRS were associated with eight and four outcomes related to mental health, respectively. Two highly pleiotropic and 10 SCZ-associated SNPs were associated with several structural brain phenotypes. Taken together, these findings indicated that GRSs of highly pleiotropic SNPs and predominantly schizophrenia-associated SNPs have partly distinct associations with brain structure and outcomes related to mental health. Thus, investigating schizophrenia-specific and pleiotropic variants may improve our understanding of the neurobiology of neuropsychiatric disorders.

## Introduction

Neuropsychiatric disorders are characterized by changes in cognition and behavior [1], and adversely impact quality of life [2]. Research suggests that up to a third of the European population have a lifetime history of neuropsychiatric features such as anxiety, insomnia, or depression [3]. Although the etiology of neuropsychiatric disorders is largely unclear, research has shown that both genetic and environmental factors influence disease development [4]. To date, research on genetic and environmental risk factors has focused largely on specific diagnoses. However, research has shown that neuropsychiatric disorders overlap in terms of their clinical characteristics [1], neural correlates [5, 6], and genetic basis [7]. Yet, research also reported disorder-specific features that may drive the development of distinct conditions [5, 8].

**Fig. 1:**
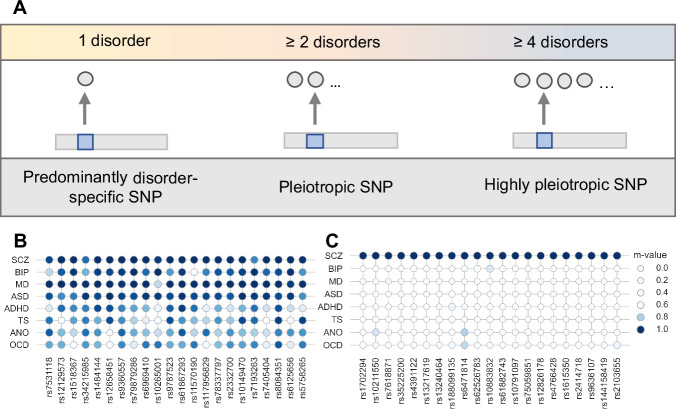
Outline of the degree of pleiotropy for genetic variants for neuropsychiatric disorders. In the second PGC cross-disorder GWAS meta-analysis across eight neuropsychiatric disorders, 146 independent lead SNPs showed genome-wide significant associations [8]. **A** These variants can be categorized into SNPs that were: (i) predominantly associated with a single disorder, (ii) associated with at least two disorders (pleiotropic SNP), or (iii) associated with at least four disorders (highly pleiotropic SNP). **B** provides an overview about the *m*-values of the 22 highly pleiotropic SNPs investigated in the present study, while **C** shows the *m*-values of the 21 predominantly SCZ-associated SNPs. We note that the term ‘predominantly’ has been added to acknowledge that while no statistical evidence has yet been generated, potential associations with additional disorders might be present. Furthermore, this figure illustrates the potential pleiotropic associations across neuropsychiatric disorders of an SNP as denoted in [8], whereby different causal scenarios are conceivable [102]. ADHD attention deficit hyperactivity disorder, ANO anorexia nervosa, ASD autism spectrum disorder, BIP bipolar disorder, GWAS genome-wide association study, MD major depression, OCD obsessive compulsive disorder, PGC Psychiatric Genomics Consortium, SCZ schizophrenia, SNP single-nucleotide polymorphism, TS Tourette’s syndrome.

Genome-wide association studies (GWAS) of neuropsychiatric disorders have demonstrated both genetic correlations [7, 8] and a high degree of genetic pleiotropy across disorders [8–10]. For example, the second cross-disorder GWAS meta-analysis by the Psychiatric Genomics Consortium (PGC-CDG2) [8], which comprised more than 230,000 patients with attention deficit hyperactivity disorder (ADHD), anorexia nervosa (ANO), autism spectrum disorder (ASD), bipolar disorder (BIP), major depression (MD), obsessive compulsive disorder (OCD), schizophrenia (SCZ), or Tourette’s syndrome (TS), identified 146 independent lead SNPs at 136 genome-wide significant loci. Of note, the PGC-CDG2 used a subset-based GWAS meta-analysis framework that was specifically designed for the investigation of heterogeneous disorders [11]. Briefly, this method allows for some disorders to have no effect and identifies the subset of disorders that yields the best meta-analyzed *z*-score [11]. The PGC-CDG2 suggested that the 146 lead SNPs have a variable degree of pleiotropy across neuropsychiatric disorders (see Fig. 1A for a schematic illustration). 23 of the lead SNPs were associated with at least four disorders, and were thus considered highly pleiotropic. In addition to these highly pleiotropic SNPs, 22 SNPs were associated predominantly with SCZ, with no reported association with any of the remaining seven neuropsychiatric disorders [8].

Follow up studies of these GWAS results have focused primarily on selected, highly pleiotropic SNPs that have been putatively mapped to genes (e.g., [8, 12, 13]). For example, rs8084351, which was associated with all eight investigated neuropsychiatric disorders, is located in an intron of the netrin-1 receptor gene *DCC*, which has been implicated in neurodevelopmental pathways via its role in promoting axon guidance [13, 14]. In addition, rs7193263, which was associated with seven of the eight investigated neuropsychiatric disorders, is located in an intron of the gene *RBFOX1*, which regulates splicing during neuronal development [15], and has been associated with aggressive and fear-related behaviors [12, 16], which occur in several neuropsychiatric disorders [17, 18]. Highly pleiotropic SNPs have thus been associated with neurobiological processes that increase the risk of general psychopathology and brain-related traits that may influence susceptibility to, and the clinical presentation of, several neuropsychiatric disorders [8].

With regard to predominantly SCZ-associated SNPs, the majority of loci were also identified in the most recent GWAS meta-analysis of SCZ by the PGC (PGC-SCZ3) [19]. In particular, rs75059851 located in an intron of the *IGSF9B* gene was suggested to be predominantly associated with SCZ by a previous study [20]. The *IGSF9B* gene is considered to encode an inhibitory synaptic cell adhesion molecule [21]. Recent research suggested that *IGSF9B* is involved in the maturation and maintenance of inhibitory synapses [21]. The functional mechanisms of the *IGSF9B* gene are largely unknown, yet it might be presumed that the predominantly SCZ-specific SNP rs75059851 together with further polymorphisms at the *IGSF9B* genetic locus lead to dysfunctional organization of inhibitory synapses in the brain [20]. Notably, recent research highlights a particular role of synaptic dysfunctions in the pathogenesis of SCZ [19, 22].

Both highly pleiotropic and predominantly SCZ-associated genetic variants might influence the susceptibility to neuropsychiatric disorders through brain-related traits [8, 23, 24]. Structural features of the brain, such as subcortical volume, cortical thickness (CT), and surface area (SA), represent potential intermediate phenotypes in the pathogenesis of neuropsychiatric disorders [23, 24]. Furthermore, the genetic architecture of structural brain phenotypes partially overlaps with that of a wide range of neuropsychiatric disorders [25–27], indicating that a substantial number of genetic variants for neuropsychiatric disorders are also associated with structural brain phenotypes. Further suggested traits in the pathway from genetic variants to disease development are outcomes related to mental health such as irritability [28], loneliness [29], and mood swings [30] for that patients with neuropsychiatric disorders show more pronounced changes [31]. Studying associations between genetic variants for neuropsychiatric disorders and brain structure, as well as outcomes related to mental health may therefore forward an increased understanding of their underlying neurobiological processes.

The effect sizes of multiple genetic variants that have been identified in the GWAS of neuropsychiatric disorders can be aggregated into one single score, which provides an estimate of an individual’s genetic predisposition to a specific trait [32]. These scores are referred to as genetic risk scores (GRS) in cases where the effect sizes of a limited number of SNPs were summarized, and as polygenic risk scores (PRS) in cases where effect sizes of SNPs across the genome were aggregated [33, 34]. Of note, GRSs that are based on (a limited number of) genome-wide significant SNPs typically have a lower predictive ability, but provide information on the precise genetic and biological mechanisms underlying a specific phenotype [35]. Research has shown that PRSs for neuropsychiatric disorders were associated with subtle structural changes in the brain [36–38] as well as outcomes related to mental health [39]. Herein, most studies calculated PRSs using effect sizes from the GWAS of specific or across neuropsychiatric disorders and did not differentiate between pleiotropic and disease-specific genetic variants. However, analyses that differentiate these two groups may advance our understanding of shared and disorder-specific neural correlates of neuropsychiatric disorders.

To address this, the aim of the present study was to investigate neural correlates of selected sets of SNPs, namely, highly pleiotropic and likewise predominantly SCZ-associated SNPs. Associations were examined between both selected structural brain phenotypes and outcomes related to mental health and: 1) a GRS for highly pleiotropic SNPs; and 2) a GRS for predominantly SCZ-associated SNPs from the PGC-CDG2. The analyses were performed using large-scale data from the UK Biobank (UKBB) [40]. In secondary analyses, the individual SNPs were annotated with GWAS summary statistics of structural brain phenotypes from the Enhancing Neuro Imaging Genetics through Meta-Analysis (ENIGMA) and Cohorts for Health and Aging Research in Genetic Epidemiology (CHARGE) consortia [41–43].

The present analyses were designed to test the following three main hypotheses. First the GRS of highly pleiotropic SNPs is associated with brain regions that are commonly affected in multiple neuropsychiatric disorders, such as the anterior cingulate and the insula [6, 44], whereas the GRS of predominantly SCZ-associated SNPs is associated with brain regions that are particularly implicated in SCZ, such as the frontal and temporal regions [44, 45]. Second, the GRS of highly pleiotropic SNPs is associated with outcomes related to mental health that are common to multiple neuropsychiatric disorders [31, 46]. Third, several individual SNPs show significant associations with brain structure.

## Materials and methods

### Study design

**Fig. 2:**
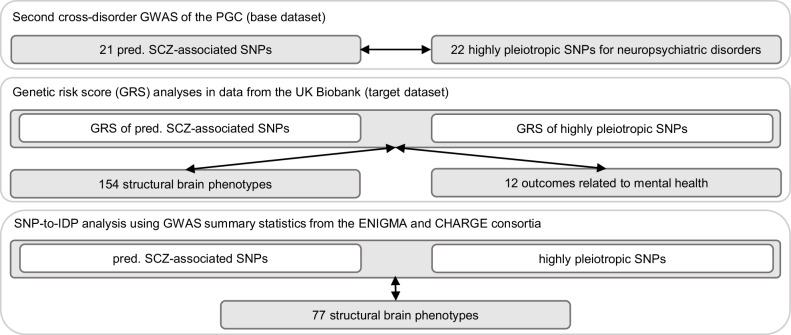
Schematic overview of the study design. Schematic overview of association analyses of highly pleiotropic (i.e., associated with at least four neuropsychiatric disorders) and predominantly SCZ-associated GRS/SNPs with brain structure and outcomes related to mental health. GRS genetic risk score, GWAS genome-wide association study, IDP image-derived phenotypes, PGC Psychiatric Genomics Consortium, pred. predominantly, SCZ schizophrenia, SNP single-nucleotide polymorphism.

The present study was performed in two steps (Fig. 2). First, the GRS of highly pleiotropic SNPs and the GRS of predominantly SCZ-associated SNPs (see section Genetic risk scores) were tested in data from the UKBB for associations with: (i) 154 image-derived phenotypes (IDPs) (see section Image-derived phenotypes); and (ii) 12 outcomes related to mental health (see section Outcomes related to mental health). Second, GWAS summary statistics of structural brain phenotypes from the ENIGMA and CHARGE consortia were used to analyze associations between individual highly pleiotropic and predominantly SCZ-associated SNPs and 77 IDPs. This set of IDPs resembled the set of IDPs in (i), but incorporated bilateral measurements. The SNP-to-IDP analysis assessed whether individual SNPs show significant associations with brain structures, thus rendering them of particular relevance for future analysis. The GWAS summary statistics of structural brain phenotypes used for this analysis were performed in samples from the UKBB and further cohorts and thus, allow to generate insights into associations between the SNPs and brain structure in a more heterogeneous sample.

### Participants

The present analyses were performed using the data from the UKBB study, which is a population-based cohort of adults aged 40–69 years at recruitment [40]. UKBB assessments took place at 22 study sites across the UK between 2006 until today. Collected data included information on medical history, mental health outcomes, and lifestyle. Data on genetic factors and brain structure were obtained via blood sampling and magnetic resonance imaging (MRI), respectively. The UKBB study was approved by the North West Multi-centre Research Ethics Committee (MREC, reference number 11/NW/0382). The ethics statement for the UKBB is available at https://www.ukbiobank.ac.uk/learn-more-about-uk-biobank/about-us/ethics. All UKBB participants provided written informed consent [40]. The present study was conducted under UKBB Application Number 41655.

The present analyses were restricted to UKBB participants of White British ancestry for whom genotype and MRI data of the brain were available. Of these, *n* = 1127 individuals were excluded due to the presence of a diagnosis that impacted the central nervous system, as derived from the 10th version of the International Statistical Classification of Diseases and Related Health Problems (ICD-10) (UKBB data field: 41270). These diagnoses included cerebrovascular- or neurodegenerative disease (A80–89, C70–72, F00–09, G00–14, G20–26, G30–32, G35–G37, I60–69, S06–09, T90, Q00–Q07, and Q90–Q99). After quality control of the genetic and brain imaging data, a subsample of *n* = 28,952 individuals remained eligible for the analyses (mean age 63.8 years, standard deviation (SD) = 7.4 years; 46.9% males).

### Materials

#### Genotyping, imputation, and quality control of the genetic data

Deoxyribonucleic acid (DNA) samples were extracted from peripheral blood cells by the UKBB study team. Genotyping was performed by the Affymetrix Research Services Laboratory using the Applied Biosystems UK BiLEVE Axiom® Array or the Applied Biosystems UKBB Axiom® Array [40].

The present analyses were performed using the imputed genotype datasets provided by the UKBB (https://biobank.ndph.ox.ac.uk/ukb/label.cgi?id=100319), which are based on the human reference assembly GRCh37. We performed standard genetic quality control using PLINK 1.9 and 2.0 [47] (see Supplementary Information for details). To correct for population stratification in further analyses, the first 10 genetic principal components (PC1–10) were calculated using PLINK 2.0. As we investigated samples with self-reported White British ancestry in the present study, we calculated the PCs ourselves and did not use the PCs provided by the UKBB.

#### Genetic risk scores

For each individual from the UKBB subsample (target dataset), the GRS of highly pleiotropic SNPs (PleioPsych-GRS) and predominantly SCZ-associated SNPs (SCZ-GRS) were computed using PRSice (v2.3.5) [48] by accumulating the weighted effect of SNPs derived from the PGC-CDG2 summary statistics [8] (base dataset). For the present analysis, we used the summary statistics that did not include subjects of the 23andMe cohort. The PRSice options of standard *p*-value thresholding and clumping were omitted since the highly pleiotropic and predominantly SCZ-associated SNPs were lead SNPs of linkage disequilibrium (LD) independent loci with genome-wide significant associations. For all subsequent analyses, *z*-score standardized GRS were used.

The definition of the highly pleiotropic and predominantly SCZ-associated SNPs was derived from the PGC-CDG2 [8]. Here, for each SNP, its associations with the individual disorders were provided by the *m*-value (cf. Table S3.2 in [8]). A value of *m* ≥ 0.8 can be considered evidence for an association of one SNP with one disorder, whereas *m* < 0.8 can be interpreted as ambiguous (cf. Figure 1 in [49]). Of note, the *m*-values required for the definition of the highly pleiotropic and predominantly SCZ-associated SNPs were only provided for the 146 genome-wide significant SNPs in [8], so that the generation and analysis of GRS using SNPs with broader *p*-value thresholds were not possible based on the data provided by the PGC-CDG2 [8] (cf. Limitations).

A highly pleiotropic SNP has shown an association with at least four neuropsychiatric disorders in the PGC-CDG2 (*m* ≥ 0.9 for at least four disorders; Fig. 1A) [8]. A predominantly SCZ-associated SNP presented a value of *m* ≥ 0.9 [8] for SCZ but values of *m* < 0.8 for the other seven neuropsychiatric disorders included in the PGC-CDG2 (Fig. 1A). The latter cut-off ensured that the SNPs were predominantly associated with SCZ.

From the 23 highly pleiotropic SNPs, the PleioPsych-GRS was calculated by aggregating the weighted effect of 22 SNPs (Fig. 1B; Table S1). The palindromic SNP rs11688767 was excluded. This SNP has a minor allele frequency (MAF) above 40%, and therefore allelic mismatches between base and target datasets could not be resolved.

From the 22 predominantly SCZ-associated SNPs, the SCZ-GRS was calculated as a weighted accumulated score of 21 SNPs (Fig. 1C; Table S2). The palindromic SNP rs2801578 (MAF > 40%) was excluded. Furthermore, rs13217619 was replaced by rs34718920 (LD: *r*^*2*^ = 1 in Utah residents with ancestry from Northern and Western Europe (CEU)) using the LDproxy tool [50], since the former SNP was not covered by the PGC-CDG2 summary statistics without 23andMe subjects [8].

To evaluate the sensitivity of effect sizes, we additionally computed the SCZ-GRS based on the effect sizes of PGC-SCZ3 [19] (see Supplementary Information for more details).

#### Brain structural image acquisition and preprocessing

The UKBB study team collected brain MRI data at four sites (Bristol, Newcastle, Cheadle, and Reading), as described elsewhere [51, 52]. In the present analyses, we preprocessed the T1-weighted MRI images using the FreeSurfer (v6) pipeline, as implemented in fMRIprep locally [53] (see Supplementary Information).

#### Image-derived phenotypes

FreeSurfer also allowed the extraction of the following: average CT per hemisphere; 2 × 34 unilateral regional CT measures, as delineated by the Desikan-Killiany atlas [54]; total SA per hemisphere, 2 × 34 unilateral regional SA measures; and 2 × 7 unilateral subcortical volume measures. This resulted in a total of 154 unilateral IDPs (Table S3). For quality reasons, IDPs that deviated more than 3 SD from the mean were excluded, and each IDP was then normalized to derive *z*-scores.

#### Outcomes related to mental health

In the present study, we focused on 12 outcomes related to mental health as the corresponding questionnaire was completed by the majority of participants from the UKBB cohort. The UKBB category ‘mental health outcomes’ included 12 factors (UKBB data field: 1920–2030): mood swings, miserableness, irritability, sensitivity / hurt feelings, fed-up feelings, nervous feelings, worrier / anxious feelings, tense feelings / highly strung, worry too long after embarrassment, suffer from nerves, loneliness, and guilty feelings. These factors were assessed using binary outcome items. The present analysis incorporated data on ‘yes’ and ‘no’ responses, but eliminated data on ‘do not know’ and ‘prefer not to answer’ responses.

### Statistical analysis

#### Associations between genetic risk scores and brain structure

Associations between the PleioPsych-GRS and the SCZ-GRS and the 154 IDPs were tested using multiple linear regression models, as implemented in the Python’s statsmodels library [55] while controlling for age at MRI visit (UKBB data field: 31-2.0), age^2^, and sex (UKBB data field: 21003-2.0). Intracranial volume was included as a covariate for SA and subcortical volume measures, as in previous ENIGMA case-control MRI studies (cf. [56–61]).

To assure statistical robustness, the analysis was repeated using an expanded set of covariates. These comprised: (i) interaction of sex and age; (ii) dummy assessment center variables (UKBB datafield: 54); (iii) Euler number [62] as a measure of image reconstruction quality; (iv) 3-dimensional head positions while scanning (UKBB datafields: 25756-2.0, 25757-2.0, 25758-2.0); and (v) PC1–10. In the analysis with and without the expanded set of covariates, false discovery rate (FDR) multiple testing correction using the Benjamini-Hochberg procedure [63] was performed across all 154 IDPs for each GRS. Statistical significance was set at *p*_FDR_ < 0.05.

Furthermore, as the PGC-CDG2 [8] included MD cases and controls from the UKBB study [64], sensitivity analyses were conducted. Herein, we excluded samples with diagnosed and self-reported depression (see Supplementary Information).

#### Associations between the genetic risk scores and outcomes related to mental health

Associations between the PleioPsych-GRS and the SCZ-GRS and the 12 outcomes related to mental health were tested using logistic regression, while controlling for sex, age, and age^2^. For each GRS, correction for multiple testing was performed across all outcomes using the Benjamini-Hochberg procedure. Results were considered significant at *p*_FDR_ < 0.05. Again, the analysis was repeated correcting for the interaction of sex and age, dummy assessment center variables (UKBB datafield: 54), and PC1–10. In addition, as presented above, a sensitivity analysis excluding samples with MD was conducted.

#### Associations between single-nucleotide polymorphisms and brain structure

Testing was performed for associations between each highly pleiotropic and each predominantly SCZ-associated SNP and 77 bilateral IDPs averaged across right and left hemispheres, as shown in Table S3. This SNP-to-IDP analysis was performed using the summary statistics of large-scale GWAS of structural brain phenotypes from the ENIGMA and CHARGE consortia [41–43]. These studies were approved by the respective ethics committees and informed consent was obtained for all participants as described elsewhere [41–43].

These summary statistics did not encompass rs117956829 (highly pleiotropic SNP) or rs10211550, rs188099135, and rs12826178 (predominantly SCZ-associated SNPs). Using the LDproxy tool [50], rs10211550 was replaced by rs11891750 (LD: *r*^*2*^ = 0.8 in CEU) and rs188099135 was replaced by rs11780834 (LD: *r*^*2*^ = 1 in CEU). For the remaining SNPs, no proxy SNP with sufficiently high LD (*r*^2^ > 0.6 in CEU) was found. Thus, 21 highly pleiotropic SNPs and 20 SCZ-associated SNPs were investigated. Again, the Benjamini-Hochberg procedure was used to correct for multiple testing. This was performed separately for the two groups of SNPs and results were considered significant at *p*_FDR_ < 0.05.

## Results

### Associations between genetic risk scores and brain structure

**Fig. 3:**
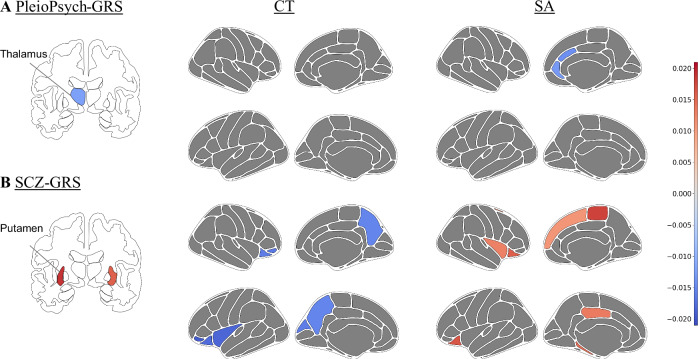
Association between the genetic risk scores and structural brain phenotypes in the UK biobank cohort. Associations between the brain volume (left column), cortical thickness (CT, middle column), and surface area (SA, right column) and **A** the genetic risk score of 22 highly pleiotropic SNPs (PleioPsych-GRS) and **B** the genetic risk score of 21 predominantly SCZ-associated SNPs (SCZ-GRS). Associations with *p*_uncorrected_ < 0.01 are depicted. Red color denotes positive, whereas blue color denotes negative, standardized beta coefficients. CT cortical thickness, GRS genetic risk score, SA surface area, SCZ schizophrenia.

For the PleioPsych-GRS, no significant association with structural brain phenotypes was found after correction for multiple testing. Nevertheless, nominally significant negative associations of the PleioPsych-GRS were found with the volume of the left thalamus, and with the SA in right caudal and rostral anterior cingulate regions (all *p*_uncorrected_ < 0.01; Table 1, Fig. 3A). These associations remained nominally significant when the extended set of covariates was used (Table S4).

**Table 1:** Associations between the genetic risk scores and structural brain phenotypes in the UK biobank cohort.

GRS	IDP	L/R	Vol./CT/SA	*p*-value	*p*_FDR_-value	*BETA*	*CI*_*lower*_	*CI*_*upper*_
PleioPsych-GRS	thalamus	L	Vol.	0.003	0.460	−0.012	−0.021	−0.004
	caudate	R	Vol.	0.019	0.563	−0.012	−0.021	−0.002
	caudate	L	Vol.	0.022	0.563	−0.012	−0.021	−0.002
	accumbens	R	Vol.	0.027	0.583	−0.011	−0.021	−0.001
	amygdala	R	Vol.	0.037	0.600	−0.010	−0.019	−0.001
	amygdala	L	Vol.	0.039	0.600	−0.010	−0.019	−0.001
	precentral	L	CT	0.044	0.612	0.011	3.2 × 10^−04^	0.022
	caudal ACC	R	SA	0.008	0.460	−0.014	−0.025	−0.004
	rostral ACC	R	SA	0.009	0.460	−0.013	−0.023	−0.003
	pars opercularis	L	SA	0.012	0.460	−0.013	−0.024	−0.003
	rostral middle frontal	L	SA	0.030	0.583	−0.009	−0.017	−0.001
	lateral orbitofrontal	L	SA	0.048	0.622	−0.008	−0.017	5.7 × 10^−05^
SCZ-GRS	putamen	L	Vol.	<0.001	**0.008**	0.019	0.010	0.028
	putamen	R	Vol.	0.001	**0.030**	0.016	0.006	0.025
	pars orbitalis	L	CT	0.001	**0.025**	−0.019	−0.031	−0.008
	insula	L	CT	0.002	**0.033**	−0.019	−0.030	−0.007
	lateral orbitofrontal	L	CT	0.002	**0.045**	−0.018	−0.029	−0.006
	lateral orbitofrontal	L	SA	<0.001	**0.008**	0.017	0.008	0.025
	paracentral	R	SA	<0.001	**0.013**	0.018	0.008	0.027
	lateral orbitofrontal	R	SA	<0.001	**0.019**	0.016	0.007	0.025

Bold font indicates significant *p*_FDR_-values. For the PleioPsych-GRS, nominally significant associations (*p* < 0.05) are presented, whereas for the SCZ-GRS, significant associations (*p*_FDR_ < 0.05) are presented. 95% CIs are shown. Associations are presented in accordance with brain measures (Vol./CT/SA).

*ACC* anterior cingulate cortex, *CI* confidence interval, *CT* cortical thickness, *FDR* false discovery rate, *GRS* genetic risk score, *IDP* image-derived phenotype, *L* left, *R* right, *SA* surface area, *SCZ* schizophrenia, *Vol*. volume.

The SCZ-GRS showed a significant association with increased volumes in both the left and right putamen; decreased CT in the left pars orbitalis, left lateral orbitofrontal cortex, and the left insula; and increased SA in the left and right lateral orbitofrontal regions (all *p*_FDR_ < 0.05; Table 1, Fig. 3B). When using the extended set of covariates, the results remained significant, with the exception of the association with the CT of the left lateral orbitofrontal region (Table S4). However, this association remained nominally significant (data not shown).

Results of the sensitivity analyses (i) excluding samples with diagnosed and self-reported MD, and (ii) using a SCZ-GRS based on the effect sizes of PGC-SCZ3 [19] are presented in the Supplementary Information. Briefly, the sensitivity analyses showed that the GRSs were associated with similar IDPs as in our main analysis. However, sensitivity analysis (i) found that six of the eight IDPs that were implicated in the main analysis remained significantly associated with the SCZ-GRS when excluding samples with MD (Table S5). Furthermore, the left amygdala volume and left parahippocampal SA were additional significantly associated with the SCZ-GRS when excluding samples with MD. Sensitivity analysis (ii) reported 21 additional significant associations that were nominally associated in the analysis of the SCZ-GRS based on PGC-CDG2 (Table S6).

### Associations between genetic risk scores and outcomes related to mental health

The PleioPsych-GRS showed a significant association with eight of the 12 outcomes related to mental health (Table 2). The lowest *p*-values were obtained for irritability (*p*_FDR_ = 8.71 × 10^−06^; odds ratio (OR) = 1.074), fed-up feelings (*p*_FDR_ = 3.41 × 10^−04^, OR = 1.056), and tense feelings (*p*_FDR_ = 7.34 × 10^−04^; OR = 1.076). The SCZ-GRS showed a significant association with four of the 12 outcomes related to mental health. The strongest associations were found for worrier / anxious feelings (*p*_FDR_ = 5.39 × 10^−03^; OR = 1.041), sensitivity / hurt feelings (*p*_FDR_ = 2.08 × 10^−02^; OR = 1.033), and guilty feelings (*p*_FDR_ = 2.32 × 10^−02^; OR = 1.036). When using the extended set of covariates, all associations remained significant (Table S7).

**Table 2:** Associations between the genetic risk scores and outcomes related to mental health in the UK biobank cohort.

Mental health outcome	PleioPsych-GRS				SCZ-GRS			
	*p*_FDR_-value	OR	*CI*_*lower*_	*CI*_*upper*_	*p*_FDR_-value	OR	*CI*_*lower*_	*CI*_*upper*_
mood swings	**7.88** **×** **10**^**−03**^	1.039	1.014	1.065	8.87 × 10^−02^	1.024	0.999	1.049
miserableness	**9.44** **×** **10**^**−04**^	1.049	1.024	1.076	4.91 × 10^−01^	1.010	0.986	1.036
irritability	**8.71** **×** **10**^**−06**^	1.074	1.045	1.103	9.63 × 10^−01^	0.999	0.973	1.027
sensitivity/hurt feelings	**4.60** **×** **10**^**−02**^	1.028	1.004	1.053	**2.08** **×** **10**^**−02**^	1.033	1.008	1.058
fed-up feelings	**3.41** **×** **10**^**−04**^	1.056	1.030	1.083	5.49 × 10^−01^	1.008	0.983	1.034
nervous feelings	**1.39** **×** **10**^**−02**^	1.045	1.013	1.077	2.63 × 10^−01^	1.020	0.989	1.051
worrier/anxious feelings	**5.40** **×** **10**^**−03**^	1.040	1.016	1.065	**5.39** **×** **10**^**−03**^	1.041	1.016	1.066
tense feelings/highly strung	**7.34** **×** **10**^**−04**^	1.076	1.037	1.116	**2.90** **×** **10**^**−02**^	1.047	1.010	1.086
worry too long after embarrassment	1.75 × 10^−01^	1.019	0.995	1.044	5.49 × 10^−01^	1.008	0.984	1.032
suffer from nerves	2.53 × 10^−01^	1.023	0.989	1.057	5.49 × 10^−01^	0.989	0.957	1.023
loneliness	8.84 × 10^−02^	1.035	1.000	1.071	8.87 × 10^−02^	1.034	0.999	1.070
guilty feelings	8.84 × 10^−02^	1.027	1.000	1.055	**2.32** **×** **10**^**−02**^	1.036	1.009	1.064

Bold font indicates significant *p*_FDR_-values. 95% CIs are presented.

*CI* confidence interval, *GRS* genetic risk score, *FDR* false discovery rate, *OR* odds ratio, *SCZ* schizophrenia.

Again, the results of the sensitivity analyses are shown in the Supplementary Information. Thereby, nine of the 12 associations of the GRSs with outcomes related to mental health remained significant when excluding samples with diagnosed and self-reported depression (Table S8). Moreover, the SCZ-GRS based on the effect sizes of PGC-SCZ3 [19] was associated with the same outcomes related to mental health compared to our main analysis (Table S9).

### Association between single-nucleotide polymorphisms and brain structure

**Fig. 4:**
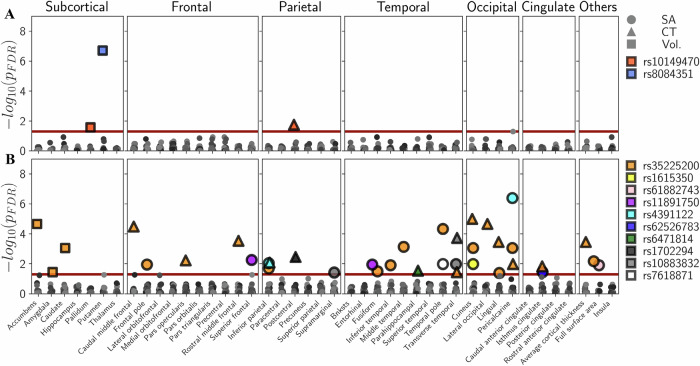
Association between highly pleiotropic and predominantly SCZ-associated single-nucleotide polymorphisms and brain structure. Associations between IDPs and **A** the highly pleiotropic SNPs and **B** the predominantly SCZ-associated SNPs using GWAS summary statistics of structural brain phenotypes from the ENIGMA and CHARGE consortia [41–43]. Significant SNP-to-IDP associations (*p*_FDR_ < 0.05, red line) are color-coded per SNP. Markers represent the three structural brain measurements (volume, CT, SA). Bnksts banks of the superior temporal sulcus, CHARGE Cohorts for Health and Aging Research in Genetic Epidemiology, CT cortical thickness, ENIGMA Enhancing Neuro Imaging Genetics through Meta-Analysis, FDR false discovery rate, GWAS genome-wide association study, IDP image-derived phenotype, SA surface area, SNP single-nucleotide polymorphism, sup. superior, temp. temporal, Vol. volume.

Among the highly pleiotropic SNPs (*n* = 21), two were significantly associated with at least one IDP. The SNP rs8084351 was significantly associated with putamen volume (*p*_FDR_ = 2.0 × 10^−07^), and rs10149470 was significantly associated with postcentral gyrus CT (*p*_FDR_ = 1.8 × 10^−02^) and pallidum volume (*p*_FDR_ = 2.7 × 10^−02^) (Fig. 4A).

Among the predominantly SCZ-associated SNPs (*n* = 20), 10 SNPs (rs35225200, rs1615350, rs61882743, rs11891750, rs4391122, rs62526783, rs6471814, rs1702294, rs10883832, and rs7618871) were significantly associated with at least one of the bilateral 77 IDPs (Fig. 4B). In particular, rs35225200 was associated with 23 IDPs including cuneus CT (*p*_FDR_ = 1.0 × 10^−05^) and accumbens volume (*p*_FDR_ = 2.2 × 10^−05^). The strongest association was found for rs4391122 and the pericalcarine SA (*p*_FDR_ = 4.1 × 10^−07^).

## Discussion

The present study investigated associations of brain structure and outcomes related to mental health with GRSs of highly pleiotropic SNPs, which were associated with at least four neuropsychiatric disorders, and GRSs of predominantly SCZ-associated SNPs. Analyses were also performed to explore associations between each individual SNP and structural brain phenotypes. While the GRS of highly pleiotropic SNPs showed no significant association with brain structure, it showed a significant association with a wide range of outcomes related to mental health. The GRS of predominantly SCZ-associated SNPs was significantly associated with brain structure, with the strongest associations being found for an increase of SA in the left lateral orbitofrontal region, and an increase in left putamen volume. Analysis of the association between brain structure and individual SNPs generated further support for structural brain associations for the predominantly SCZ-associated SNPs, but limited support for the highly pleiotropic SNPs.

### Associations between the genetic risk scores and brain structure

No significant associations were found between the PleioPsych-GRS and brain structure IDPs. A plausible hypothesis is that highly pleiotropic SNPs do not necessarily influence brain structure in the general population. Moreover, previous authors have reported genetic variants for neuropsychiatric disorders to be associated with further brain measures such as structural and functional connectivity, white matter microstructure, or neurochemistry [36, 37, 65]. Therefore, it might be hypothesized that the role of the highly pleiotropic loci could be rather observable in other brain measures. Another possible reason for the lack of any significant association between the PleioPsych-GRS and brain structure IDPs is that highly pleiotropic loci may influence more diverse neurobiological processes, such as distinct cellular subcomponents or cellular subtypes [66]. In that case, their aggregated effects would not be expected to affect specific structural brain phenotypes.

The PleioPsych-GRS did, however, show a nominally significant association with decreased left thalamic volume and SA in the right caudal and rostral anterior cingulate regions. The thalamus relays sensory information, and plays an extensive role in cognition as an integrative hub [67]. Large-scale MRI case-control studies have reported decreased thalamic volume across multiple neuropsychiatric disorders [68, 69], and the thalamus is considered one of the structural epicenters of neuropsychiatric disease [70]. Moreover, the anterior cingulate region is involved in social-emotional processing [71], and parts of the anterior cingulate have been reported to show subtle structural changes across multiple neuropsychiatric disorders [6]. To clarify the influence of the highly pleiotropic SNPs on the structure of the thalamus and the anterior cingulate region, further genomic imaging studies with larger sample sizes and a focus on these specific regions are required.

The SCZ-GRS showed a significant association with increased SA as well as decreased CT in the lateral orbitofrontal region. Notably, a case-control MRI study including 4474 individuals with SCZ observed CT thinning throughout the cortex, including the lateral orbitofrontal region in SCZ patients compared to controls [45]. The largest effect sizes, however, were found for regions of the prefrontal and temporal cortex [45]. When additionally controlling for average CT, the observation of decreased CT in prefrontal regions including the left lateral orbitofrontal region remained significant [45]. While these results support a disproportional implication of the prefrontal regions, further neuroimaging studies of SCZ underscored pronounced alterations in these regions for patients with SCZ (e.g. [72–75]). Furthermore, structural changes in the orbitofrontal region may contribute to symptoms that occur in SCZ [76–78]. In this context, research has shown that decreased CT in the orbitofrontal region was associated with negative symptoms in patients with first-episode SCZ [79]. Beyond that, the orbitofrontal region was suggested to be involved in higher cognitive functions, including emotional and reward processing [80], which is dysfunctional in patients with SCZ [31]. Together, this suggests a central role of the prefrontal cortex in the pathophysiology of SCZ.

It should be noted that previous studies also reported an association between PRS for neuropsychiatric disorders and this region. In particular, the PRS for SCZ was found to be associated with decreased CT in the right lateral orbitofrontal region [81]. Besides that, research has demonstrated significant associations between the PRS for MD and cortical complexity in the lateral orbitofrontal region [38], and between the PRS for BIP and SA in the lateral orbitofrontal region [82]. Future studies should therefore investigate whether the aggregated effect of predominantly SCZ-associated SNPs influences different measures of the lateral orbitofrontal region.

The SCZ-GRS also showed a significant association with increased left and right putamen volume. The putamen is a component of the basal ganglia, and besides its involvement in motor control, it is implicated in cognitive functions such as language and reward processing [83]. Research has demonstrated a decrease in putamen volume in patients with ADHD and MD, while an increase in putamen volume has been found in other neuropsychiatric disorders, in particular SCZ and BIP [84]. Previous authors have proposed that this increase results from an overexpression of dopaminergic neurons [84]. However, antipsychotic treatment may also have made a partial contribution to the increase in volume [85], given that putamen volume has shown significant positive associations with illness duration [86]. Further insights into the convergence of predominantly SCZ-associated genetic variants on the putamen could facilitate the identification of neurobiological processes that are specific to SCZ.

### Associations between the genetic risk scores and outcomes related to mental health

Both the PleioPsych-GRS and the SCZ-GRS showed a significant association with the mental health related outcomes worrier, sensitivity, and tense feelings. The PleioPsych-GRS also showed significant associations with mood swings, miserableness, irritability, fed-up feelings, and nervous feelings, all of which represent items from the neuroticism scale of the Eysenck Personality Questionnaire-Revised Short Form [87]. The significant associations of the PleioPsych-GRS with eight outcomes related to mental health illustrated that this GRS effects brain-related traits despite the absence of significant associations with structural brain phenotypes. Furthermore, the PleioPsych-GRS was associated with more outcomes related to mental health compared to the SCZ-GRS, which may reflect, at least in part, that these outcomes largely represent cross-disorder traits [31]. However, the 12 outcomes related to mental health that were assessed in the UKBB are limited in terms of their depth in quantifying cognitive and behavioral changes [39]. Therefore, to investigate the phenotypic manifestation of the GRSs in more detail, studies involving deep phenotyping data are required.

### Association between single-nucleotide polymorphisms and brain structure

In the present SNP-to-IDP analysis, more predominantly SCZ-associated SNPs (*n* = 10) were associated with IDPs than highly pleiotropic SNPs (*n* = 2). The implicated IDPs included SA and CT measures of the occipital and temporal cortices. This is a notable finding, since the PGC-CDG2 found non-pleiotropic loci (mainly SCZ-associated) to be enriched for occipital cortex specific genes (cf. Figure 5C in the PGC-CDG2 [8]), which has not been reported for pleiotropic loci [8]. In particular, the predominantly SCZ-associated SNP rs35225200 was associated with 23 IDPs. rs35225200 is located near the gene *SLC39A8* encoding a protein that acts in the transport of metals, which may be crucial for the pathogenesis of SCZ including neurotransmission [88, 89]. In this context, previous studies in a subset of the UKBB sample have already found that rs35225200 as well as further SNPs within 500 kb of the *SLC39A8* locus were significantly associated with structural brain phenotypes across multiple regions and measures of the brain [90].

### Limitations

When interpreting the present results, both the limitations and strengths of the approach must be considered. First, our investigations were based on data from the UKBB cohort, which represents a large sample size for the reliable detection of brain-phenotype associations [91]. However, the UKBB cohort largely comprises middle-aged to older adults from the general population [40, 92]. Future studies must therefore further elucidate our findings across the entire age spectrum and in clinical neuropsychiatric disorder cohorts. Several of the identified associations between brain structure and the SCZ-GRS were consistent with the literature, which suggests that future studies in patient cohorts might detect larger effects. The present analyses were restricted to individuals of White British ancestry, and future studies are warranted to identify similarities and differences between ancestries. Furthermore, the PGC-CDG2 [8] included MD cases and controls who had participated in the UKBB study [64]. Therefore, the base and target datasets were not mutually exclusive. Our sensitivity analyses, which excluded cases with depression based on a reconstruction of the case ascertainment of Wray et al. [64], found concordance in the effect sizes of the association between GRSs and structural brain phenotypes as well as the odds ratios of the association between GRSs and outcomes related to mental health (see Supplementary Information). Yet, the influence of a potential overlap of controls could not be assessed in the present sensitivity analyses.

Second, we explored the association between the GRSs and outcomes related to mental health provided by participants through binary responses. It is important to acknowledge the limitations of this assessment, which, due to its simplified nature and constrained response options, is susceptible to response bias [93]. Nevertheless, we point out that previous studies have demonstrated the validity of the UKBB mental health assessment (e.g., [94]). Future studies may explore the association between the GRSs and mental health outcomes using more detailed psychometric questionnaires, e.g., based on a rating scale [95].

Third, the PleioPsych-GRS and the SCZ-GRS were based on SNPs identified by the PGC-CDG2 [8]. The majority of the highly pleiotropic SNPs were associated with SCZ, BIP, MD, and ASD, and less frequently with ADHD, TS, OCD, and ANO, which was partly attributable to the limited number of cases with the latter diagnoses in the PGC-CDG2 [8]. Future cross-disorder GWASs that include more patients from these underrepresented disorder groups [74] may expand the set of highly pleiotropic and predominantly disorder-associated SNPs. Larger studies identifying more highly pleiotropic and predominantly disease-specific variants might also facilitate analyses of GRSs with a higher number of SNPs, e.g., by applying more liberal *p*-value thresholds, which was not possible in the present study due to lack of data availability. Nonetheless, we note that analyses with GRSs based on a limited number of SNPs (e.g. genome-wide significant SNPs) are widely used in the investigation of genetic phenotypes and that these GRSs can be powerful and reliable predictors (e.g. [96, 97]). In addition, the findings of our sensitivity analyses largely confirm the robustness of the results of the present study.

Fourth, brain structure is one of many intermediate phenotypes that link genetics to disease development [23, 24]. Future studies are needed to explore associations between the highly pleiotropic and predominantly SCZ-associated SNPs and additional brain metrics, such as structural and functional connectivity [98] and microstructure [37], as well as structural brain phenotypes delineated by more fine-grained brain atlases (e.g. [99]).

Lastly, causal implications underlying the association between genetic variation and brain-related traits could not be inferred. While Mendelian Randomization (MR) might be applied to address this aspect in principle, MR analyses based on a restricted number of SNPs tend to be affected by weak instrument bias [100]. Furthermore, SNPs associated with neuropsychiatric disorders show in many cases associations with additional brain-related traits [101], potentially violating the MR assumptions that genetic instruments do not influence the outcome other than through the exposure [100]. For these reasons, we did not perform MR analyses based on the present highly pleiotropic or predominantly SCZ-associated SNPs. However, further studies using functional genomic analyses may elucidate the causal association between genetic variants for neuropsychiatric disorders and structural brain phenotypes.

## Conclusion

The present study found that the GRS of highly pleiotropic SNPs, which were associated with at least four neuropsychiatric disorders in the PGC-CDG2, was significantly associated with outcomes related to mental health, but not structural brain phenotypes. In contrast, GRS of predominantly SCZ-associated SNPs showed significant positive associations, with the most statistically robust associations being found for SA of the lateral orbitofrontal region and putamen volume. Moreover, the predominantly SCZ-associated SNP rs35225200 showed significant associations with 23 structural brain phenotypes suggesting a complex role in shaping brain structure. These findings may indicate the existence of distinct neurobiological correlates for highly pleiotropic and predominantly SCZ-associated loci, and underline the importance of further studies on elucidating the underlying neurobiological processes of shared and disorder-specific genetic risk for neuropsychiatric disorders.

## Supplementary information

Supplemental Material

## Data Availability

The data from the UK Biobank can be accessed upon approval (https://www.ukbiobank.ac.uk/). The summary statistics of the second cross-disorder GWAS and the most recent GWAS of SCZ by the PGC are publicly available for all researchers (https://pgc.unc.edu/for-researchers/download-results/). The summary statistics of structural brain phenotypes by the ENIGMA-CHARGE collaboration can be accessed upon request (http://enigma.usc.edu/research/download-enigma-gwas-results/).
