# Supplementary material for: Neurobiological correlates of schizophrenia-specific and highly pleiotropic genetic risk scores for neuropsychiatric disorders
Source: Transl Psychiatry. 2025 Jul 5;15:230. doi: 10.1038/s41398-025-03440-1 (PMC12228695; doi:10.1038/s41398-025-03440-1)
Supplement: Supplementary file 1 — Supplemental Material [file 41398_2025_3440_MOESM1_ESM.pdf]

Supplementary Information

Preprocessing of UK Biobank data **2**
Genetic quality control 2
Magnetic resonance imaging data preprocessing 2
Sensitivity analyses excluding samples with diagnosed and self-reported depression **2**
Sensitivity analyses using the GRS computed based on effect sizes from a GWAS of schizophrenia **4**
Supplementary Tables **6**
Table S1 6
Table S2 9
Table S3 11
Table S4 12
Table S5 13
Table S6 14
Table S7 15
Table S8 16
Table S9 17
Supplementary Figures **18**
Figure S1 18
References **19**

Preprocessing of UK Biobank data

Genetic quality control

Individuals with any of the following were excluded: 1) a mismatch between self-reported and genetically inferred sex; 2) high rates of genotype missingness (>0.05), or 3) high individual heterozygosity rates (±3 standard deviations from the mean). A further exclusion criterion was up to and including second degree relatedness (kinship coefficient >0.088 (1)). In such cases, one individual per pair was retained.

Variants with any of the following characteristics were excluded: 1) a minor allele frequency (MAF) <0.01, 2) a variant call rate<0.95, 3) a deviation from Hardy-Weinberg Equilibrium (HWE) with $p<1\times10^{-6}$, or 4) an imputation quality score (INFO)<0.3 (2).

Magnetic resonance imaging data preprocessing

Preprocessing steps included skull-stripping, spatial normalization, brain tissue segmentation, and surface reconstruction. In addition, FreeSurfer (v6) was used to calculate the Euler number (3) as a proxy for quality of cortical reconstruction. The number was included as a covariate in the subsequent analyses. The FreeSurfer pipeline automatically segmented the volume of seven unilateral subcortical structures (amygdala, accumbens, caudate, hippocampus, pallidum, putamen, and thalamus) (4), and parcellated 34 unilateral brain regions for surface area (SA) and cortical thickness (CT), as delineated by the Desikan-Killiany atlas (5). Furthermore, total SA and average CT were extracted per hemisphere. The image-derived phenotypes (IDPs) are listed in Table S3. The total intracranial volume was segmented and included as a covariate in the analyses.

Sensitivity analyses excluding samples with diagnosed and self-reported depression

Given that the second cross-disorder genome-wide association study (GWAS) meta-analysis by the Psychiatric Genomics Consortium (PGC-CDG2) (6) comprised samples of a GWAS of major depression (MD) that were part of the interim data release of the UK Biobank (UKBB)

(7), we conducted sensitivity analyses excluding samples with self-reported and registered diagnosis of depression.

Exclusion of participants

We reconstructed the case ascertainment presented in Table S2 of (7), which comprised registered ICD-10 code for major depressive disorder, and self-reports that endorsed having seen a psychiatrist for anxiety or depression, or having experienced depression or anhedonia for a period that lasted at least two weeks. From our main study population (n=28,952), we excluded all samples with a registered ICD-10 code of F32 and F33 (UKBB datafield: 41270), all samples that stated having seen a psychiatrist for depression or anxiety (UKBB datafields: 2100-0.0 and 2100-1.0), and all samples that reported depression or anhedonia symptoms for at least two weeks (UKBB datafield: 4609-0.0 and 4609-1.0). Thereby, the self-reports of the samples' first and second assessments were taken given that the GWAS of MD (7) was based on the interim data release of the UKBB. As a result, a study population of 21,556 samples with a mean age of 64.0 years (SD=7.4 years) and 48.6% males remained for the sensitivity analyses.

Sensitivity analyses

Our sensitivity analyses investigated whether the results remained stable after excluding samples with diagnosed and self-reported depression that may have been included in the PGC-CDG2 (6). In this regard, the association analyses of the genetic risk scores (GRSs) with IDPs as well as outcomes related to mental health were repeated based on the remaining samples. Next, concordance correlation coefficients ρ of the effect sizes between the main and sensitivity analyses were computed (8).

Regarding the associations of the GRSs and IDPs, we observed concordance of effect sizes obtained in our main and sensitivity analyses (ρ=0.94) (Table S5). Yet, only six of eight associations between the GRS-SCZ and IDPs remained significant, which is likely due to the substantial reduction in sample size (Table S5). In addition, the GRS-SCZ was significantly associated with left amygdala volume and left parahippocampal SA.

Regarding the associations of the GRSs and outcomes related to mental health, we observed concordance of the odds ratios obtained in our main analysis and the sensitivity analysis (ρ=0.72) (Table S8). For the PleioPsych-GRS, five out of eight associations from the main analysis remained significant in our sensitivity analysis. For the SCZ-GRS, three additional significant associations with mood swings, miserableness, and loneliness were observed. We presume that the reduced number of significant associations with the PleioPsych-GRS may be due to the exclusion of samples with self-reported depression that, in turn, led to a lower sample size and potentially to a decrease in the variance of the outcomes related to mental health.

Sensitivity analyses using the GRS computed based on effect sizes from a GWAS of schizophrenia

Regarding the predominantly SCZ-associated single-nucleotide polymorphisms (SNPs), sensitivity analyses were conducted to investigate whether the effect sizes used for the computation of the SCZ-GRS might have affected the results of the present study. In our main analyses, the SCZ-GRS was computed based on the effect sizes from the PGC-CDG2 (6). Herein, a subset-based GWAS meta-analysis framework was used (9). Yet, to investigate the sensitivity of the origin of the effect sizes, we repeated the analyses presented in this study using a SCZ-GRS based on effect sizes taken from the most recent GWAS of SCZ by the PGC (PGC-SCZ3) (10). According to the cohort overview in Trubetskoy et al. (2022), no samples from the UKBB were included in the PGC-SCZ3.

SCZ-GRS computed based on the effect sizes from the PGC-SCZ3

The effect sizes of the 21 predominantly SCZ-associated SNPs were extracted from the core GWAS summary statistics of the PGC-SCZ3, which included samples of multiple ancestries (10). This GWAS comprised 67,390 patients with SCZ and 94,015 control samples (10). Similar to our main analyses, we excluded rs2801578 and replaced rs13217619 with rs34718920 (see Material and Methods). In the GWAS summary statistics of the PGC-SCZ3, the SNPs

rs10211550, rs11782089, and rs144158419 were not covered and hence, these SNPs were substituted using the proxy SNPs rs67657812 (Linkage Disequilibrium (LD): r^2=0.8 in Utah residents with ancestry from Northern and Western Europe (CEU)), rs7839435 (LD: r^2=1 in CEU), and rs72934586 (LD: r^2=1 in CEU), respectively, using the LDproxy tool (11).

We observed that the effect sizes of the 21 predominantly SCZ-associated SNPs derived from the PGC-SCZ3 and the respective effect sizes derived from the PGC-CDG2 were strongly correlated (Figure S1). Furthermore, in the data from the UKBB, we found that the SCZ-GRS based on PGC-SCZ3 strongly correlated with the SCZ-GRS based on PGC-CDG2 (r>0.99).

Sensitivity analyses

Both GRSs were significantly associated with similar IDPs (Table 1, Table S6). Yet, the SCZ-GRS based on PGC-SCZ3 was significantly associated with 29 IDPs. Of these, 8 IDPs were significantly and 21 IDPs were nominally associated with the SCZ-GRS based on PGC-CDG2. Furthermore, the SCZ-GRS based on PGC-SCZ3 was significantly associated with worrier (OR=1.042; p_{FDR}=0.004), sensitivity (OR=1.034; p_{FDR}=0.017), guilty feelings (OR=1.037; p_{FDR}=0.022), and tense feelings (OR=1.046; p_{FDR}=0.034) (Table S9). These outcomes related to mental health were also significantly associated with the SCZ-GRS based on PGC-CDG2 (Table 2). In conclusion, the results of the SCZ-GRS analyses based on the effect sizes of PGC-CDG2 or PGC-SCZ3 were largely comparable.

Supplementary Tables

Table S1

Variant information for the 22 highly pleiotropic single-nucleotide polymorphisms

Index	rsID	CHR	BP	EA	OA	*BETA*	CADD	RDB	Implicated disorder	VEP	Nearest gene
1	rs7531118	1	72837239	T	C	-0.035	11.35	4	ANO, ASD, MD, SCZ, TS	Regulatory region variant	*NEGR1*
2	rs12129573	1	73768366	A	C	0.053	5.32	7	ADHD, BIP, MD, SCZ	Upstream gene variant	*LRRIQ3*
3	rs1518367	2	198807015	A	T	-0.050	0.08	6	ASD, BIP, MD, SCZ	Intron variant	*PLCL1*
4	rs34215985	4	42047778	C	G	-0.052	6.84	6	ADHD, ANO, ASD, MD, SCZ, TS	Intron variant	*SLC30A9*
5	rs1484144	4	80217597	T	C	0.036	3.35	6	ADHD, ASD, BIP, MD, SCZ	Intron variant	*NAA11*
6	rs12658451	5	103904037	T	C	0.037	5.86	6	ADHD, ASD, BIP, MD, SCZ, TS	Intron variant	*NUDT12*
7	rs9360557	6	73132745	C	G	0.034	0.95	7	ADHD, ANO, ASD, BIP, MD, SCZ	Regulatory region variant	*KCNQ5*
8	rs79879286	7	24826589	C	G	0.046	2.13	2b	ASD, BIP, MD, SCZ	Regulatory region variant	*GSDME*
9	rs6969410	7	110069015	T	G	0.051	2.13	7	ASD, BIP, MD, OCD, SCZ	Downstream gene variant	*LRRN3*
10	rs10265001	7	140665521	C	G	-0.053	8.94	5	ASD, BIP, OCD, SCZ	Intergenic variant	*BRAF*
11	rs9787523	10	106460460	T	C	0.035	9.77	7	ADHD, ASD, MD, SCZ, TS	Intron variant	*SORCS3*

Continues on the next page

Table S1 continued

Index	rsID	CHR	BP	EA	OA	*BETA*	CADD	RDB	Implicated disorder	VEP	Nearest gene
12	rs61867293	10	106563924	T	C	-0.050	8.24	7	ADHD, ANO, ASD, BIP, MD, SCZ	Intron variant	*SORCS3*
13	rs11570190	11	57560452	A	C	-0.034	6.11	1b	ADHD, ASD, MD, OCD, SCZ	Intron variant	*CTNND1*
14	rs117956829	11	89339666	A	G	0.109	0.97	6	ANO, ASD, BIP, MD, SCZ	Regulatory region variant	*TRIM77*
15	rs78337797	12	23987925	T	G	0.042	2.21	7	ASD, BIP, MD, SCZ	Intron variant	*SOX5*
16	rs2332700	14	72417326	C	G	0.047	4.26	7	ASD, BIP, MD, SCZ	Intron variant	*RGS6*
17	rs10149470	14	104017953	A	G	-0.039	0.47	2b	ASD, BIP, MD, SCZ, TS	Upstream gene variant	*KLC1*
18	rs7193263	16	6315880	A	G	-0.039	0.14	7	ADHD, ASD, BIP, MD, OCD, SCZ, TS	Intron variant	*RBFOX1*
19	rs7405404	16	13749859	T	C	0.070	1.33	7	ASD, BIP, MD, SCZ	Intergenic variant	*ERCC4*
20	rs8084351	18	50726559	A	G	0.044	4.03	6	ADHD, ANO, ASD, BIP, MD, OCD, SCZ, TS	Intron variant	*DCC*
21	rs6125656	20	48090779	A	G	0.063	0.93	7	ASD, BIP, MD, SCZ	Intron variant	*KCNB1*
22	rs5758265	22	41617897	A	G	0.073	7.61	7	MD, OCD, SCZ, TS	Intron variant	*L3MBTL2*

Note. Variant information adapted from the second cross-disorder genome-wide association study of the Psychiatric Genomics Consortium (6), with the exception of rs11688767 due to non-inferable allele ambiguity. Information on the CADD score, the VEP, and the nearest gene were taken from the Open Targets Genetics portal version 22.10 (12,13). The CADD score indicates potential deleterious effects, and the RDB score (14) indicates regulatory functionality. *Abbreviations*. ADHD, attention deficit hyperactivity disorder; ANO, anorexia nervosa; ASD, autism spectrum disorder;

BETA, effect size; BIP, bipolar disorder; BP, base-pair position in GRCh37; CADD, combined annotation dependent depletion score; CHR, chromosome; EA, effect allele; MD, major depression; OA, other allele; OCD, obsessive compulsive disorder; RDB, RegulomeDB score; SCZ, schizophrenia; TS, Tourette's syndrome; VEP, variant effect predictor.

Table S2

Variant information for the 21 predominantly SCZ-associated single-nucleotide polymorphisms

Index	rsID	CHR	BP	EA	OA	*BETA*	CADD	RDB	Implicated disorder	VEP	Nearest gene
1	rs1702294	1	98501984	T	C	-0.115	0.13	n.a.	SCZ	Intron variant	*DPYD*
2	rs10211550	2	198383299	T	G	-0.069	0.05	6	SCZ	Intron variant	*HSPD1*
3	rs7618871	3	136400420	T	G	0.070	0.19	6	SCZ	Intron variant	*STAG1*
4	rs35225200	4	103146888	A	C	-0.135	2.40	7	SCZ	Intergenic variant	*SLC39A8*
5	rs4391122	5	60598543	A	G	-0.078	4.56	5	SCZ	Intergenic variant	*ZSWIM6*
6	rs34718920	6	27783941	T	C	-0.213	3.62	3a	SCZ	Upstream gene variant	*H2BC14*
7	rs13240464	7	110898915	T	C	0.075	0.72	6	SCZ	Intron variant	*LRRN3*
8	rs188099135[1]	8	27411792	A	G	-0.065	2.12	6	SCZ	Intergenic variant	*CLU*
9	rs6471814	8	60697874	T	G	0.064	1.26	7	SCZ	Intergenic variant	*CA8*
10	rs62526783	8	111471166	A	G	0.065	2.51	5	SCZ	Intergenic variant	*KCNV1*
11	rs10883832	10	104871279	T	G	0.161	12.48	1f	SCZ	Intron variant	*RPEL1*
12	rs61882743	11	46548754	C	G	-0.069	0.22	3a	SCZ	Intron variant	*AMBRA1*
13	rs10791097	11	130718630	T	G	0.077	9.19	5	SCZ	Intron variant	*SNX19*
14	rs75059851	11	133822569	A	G	0.089	6.03	3a	SCZ	Intron variant	*IGSF9B*
15	rs12826178	12	57622371	T	G	-0.168	2.50	6	SCZ	Upstream gene variant	*SHMT2*
16	rs4766428	12	110723245	T	C	0.064	2.01	5	SCZ	Intron variant	*ATP2A2*
17	rs1615350	12	123650335	T	C	-0.085	7.91	5	SCZ	Intron variant	*PITPNM2*
18	rs2414718	15	61863133	A	G	0.068	0.23	6	SCZ	Intron variant	*RORA*
19	rs9636107	18	53200117	A	G	-0.080	5.05	5	SCZ	Intron variant	*TCF4*
20	rs144158419	18	53554733	T	C	-0.142	0.65	7	SCZ	Intron variant	*TCF4*
21	rs2103655	20	37425958	A	G	0.077	2.48	n.a.	SCZ	Intergenic variant	*PPP1R16B*

Note. Variant information adapted from (6). rs2801578 was excluded due to non-inferable allele ambiguity. SNP rs13217619 was replaced by rs34718920 as it was not covered in the GWAS summary statistics (6). Resources used for SNP annotation are described in the legend of Table S1. [1]The rsID rs188099135 has been merged into the rsID rs11782089. While the former rsID has been listed by the second cross-disorder GWAS by the Psychiatric Genomics Consortium (6), the latter rsID was used for the analyses and database requests presented in this study. *Abbreviations*. BETA, effect size; BP, basepair position in GRCh37; CADD, combined annotation dependent depletion score; CHR, chromosome; EA, effect allele; GWAS, genome-wide association study; n.a., not available; OA, other allele; RDB, RegulomeDB score; SCZ, schizophrenia; VEP, variant effect predictor.

Table S3

Image-derived phenotypes

Brain measures	Number of IDPs (uni-/bi-lateral)	Brain structural phenotypes
Volume	14/7	Volumes of the following seven structures: accumbens, amygdala, caudate nucleus, hippocampus, pallidum, putamen, and thalamus
Regional CT, SA	136/68	CT and SA measures of the following Desikan-Killiany regions: banks of the superior temporal sulcus, caudal anterior cingulate, caudal middle frontal, cuneus, entorhinal, frontal pole, fusiform, inferior parietal, inferior temporal, insula, isthmus cingulate, lateral occipital, lateral orbitofrontal, lingual, medial orbitofrontal, middle temporal, paracentral, parahippocampal, pars opercularis, pars orbitalis, pars triangularis, pericalcarine, posterior cingulate, precuneus, precentral, postcentral, rostral anterior cingulate, rostral middle frontal, superior frontal, superior parietal, superior temporal, supramarginal, temporal pole, transverse temporal
CT, SA per hemisphere/whole-brain	4/2	Average CT, total SA

Note. Image-derived phenotypes that were considered in the present study. The genetic risk score analyses included 154 unilateral IDPs. The exploratory SNP-to-IDP analyses used bilateral IDPs as given by the ENIGMA-CHARGE GWAS (15–17) resulting in 77 IDPs. *Abbreviations*. CT, cortical thickness; IDPs, image-derived phenotypes; SA, surface area.

Table S4

Associations between genetic risk scores and brain structure in the UK Biobank, as calculated using the extended set of covariates

GRS	IDP	L/R	Vol./CT/SA	p-value	p_{FDR}-value	$BETA$	SE	CI_{lower}	CI_{upper}
Pleio-Psych-GRS	thalamus	L	Vol.	0.002	0.357	-0.013	0.004	-0.021	-0.005
	caudate	R	Vol.	0.023	0.490	-0.011	0.005	-0.021	-0.002
	caudate	L	Vol.	0.028	0.490	-0.011	0.005	-0.021	-0.001
	accumbens	R	Vol.	0.029	0.490	-0.011	0.005	-0.021	-0.001
	amygdala	L	Vol.	0.029	0.490	-0.010	0.005	-0.020	-0.001
	amygdala	R	Vol.	0.032	0.490	-0.010	0.005	-0.019	-0.001
	thalamus	R	Vol.	0.043	0.605	-0.008	0.004	-0.016	2.4×10^{-04}
	caudal ACC	R	SA	0.010	0.490	-0.014	0.005	-0.025	-0.003
	rostral ACC	R	SA	0.012	0.490	-0.013	0.005	-0.023	-0.003
	pars opercularis	L	SA	0.015	0.490	-0.013	0.005	-0.023	-0.002
	rostral middle frontal	L	SA	0.032	0.490	-0.009	0.004	-0.017	-0.001
SCZ-GRS	putamen	L	Vol.	<0.001	**0.006**	0.020	0.005	0.010	0.029
	putamen	R	Vol.	0.001	**0.022**	0.016	0.005	0.007	0.026
	pars orbitalis	L	CT	0.001	**0.027**	-0.019	0.006	-0.030	-0.008
	insula	L	CT	0.002	**0.037**	-0.018	0.006	-0.030	-0.007
	lateral orbito-frontal	L	SA	<0.001	**0.007**	0.017	0.004	0.008	0.025
	lateral orbito-frontal	R	SA	<0.001	**0.016**	0.016	0.005	0.007	0.025
	paracentral	R	SA	<0.001	**0.016**	0.017	0.005	0.008	0.027

Note. Results of testing for associations between the GRSs and brain structural phenotypes using an extended set of covariates. Bold font indicates significant p_{FDR}-values. For the PleioPsych-GRS, nominally significant associations (p<0.05) are presented. For the SCZ-GRS, significant associations (p_{FDR}<0.05) are presented. 95% CIs were given. *Abbreviations*. ACC, anterior cingulate cortex; CI, confidence interval; CT, cortical thickness; FDR, false-discovery rate; GRS, genetic risk score; IDP, image-derived phenotype; L, left; R, right; SA, surface area; SCZ, schizophrenia; SE, standard error; Vol., volume.

Table S5

Associations between genetic risk scores and brain structure in the UK Biobank excluding samples with registered ICD-10 diagnosis and self-report of MD

GRS	IDP	L/R	Vol./CT/SA.	p-value	p_{FDR}-value	*BETA*	*SE*	CI_{lower}	CI_{upper}
Pleio-Psych-GRS	thalamus	L	Vol.	0.004	0.568	-0.014	0.005	-0.024	-0.005
	caudate	R	Vol.	0.041	0.736	-0.012	0.006	-0.023	0.0005
	thalamus	R	Vol.	0.041	0.736	-0.009	0.005	-0.019	0.0004
	precentral	L	CT	0.029	0.736	0.014	0.007	0.001	0.027
	isthmus cingulate	R	CT	0.034	0.736	0.014	0.007	0.001	0.027
	pars triangularis	R	SA	0.007	0.575	-0.016	0.006	-0.028	-0.004
	pars opercularis	L	SA	0.012	0.633	-0.015	0.006	-0.027	-0.003
	rostral middle frontal	L	SA	0.044	0.736	-0.010	0.005	-0.020	0.0003
SCZ-GRS	putamen	L	Vol.	<0.001	**0.003**	0.024	0.006	0.013	0.035
	putamen	R	Vol.	0.001	**0.020**	0.019	0.006	0.008	0.030
	amygdala	L	Vol.	0.002	**0.047**	0.017	0.006	0.006	0.028
	pars orbitalis	L	CT	<0.001	**0.020**	-0.024	0.007	-0.037	-0.011
	insula	L	CT	0.002	**0.047**	-0.021	0.007	-0.034	-0.007
	lateral orbitofrontal	L	SA	<0.001	**0.010**	0.019	0.005	0.009	0.029
	lateral orbitofrontal	R	SA	0.002	**0.047**	0.017	0.005	0.006	0.027
	parahippocampal	L	SA	0.002	**0.047**	0.018	0.006	0.007	0.030

Note. Results of testing for associations between the GRSs and brain structural phenotypes excluding samples with registered ICD-10 diagnosis and self-report of depression. Bold font indicates significant p_{FDR}-values. For the PleioPsych-GRS, nominally significant associations (p<0.05) are presented. For the SCZ-GRS, significant associations (p_{FDR}<0.05) are presented. 95% CIs were given. *Abbreviations*. ACC, anterior cingulate cortex; CI, confidence interval; CT, cortical thickness; FDR, false-discovery rate; GRS, genetic risk score; IDP, image-derived phenotype; L, left; MD, major depression; R, right; SA, surface area; SCZ, schizophrenia; SE, standard error; Vol., volume.

Table S6

Associations between the SCZ-GRS based on effect sizes of a GWAS of SCZ and brain structure

IDP	L/ R	Vol./ CT/ SA	p-value	p_{FDR}-value	$BETA$	SE	CI_{lower}	CI_{upper}
putamen	L	Vol.	<0.001	0.001	0.021	0.005	0.012	0.030
putamen	R	Vol.	<0.001	0.007	0.018	0.005	0.008	0.027
insula	L	CT	0.001	0.014	-0.020	0.006	-0.031	-0.009
pars orbitalis	L	CT	0.001	0.014	-0.020	0.006	-0.031	-0.008
lateral orbitofrontal	L	CT	0.001	0.014	-0.020	0.006	-0.031	-0.008
lateral orbitofrontal	R	CT	0.002	0.027	-0.019	0.006	-0.030	-0.007
paracentral	L	CT	0.002	0.027	-0.018	0.006	-0.029	-0.006
cuneus	L	CT	0.002	0.027	-0.018	0.006	-0.029	-0.007
pars orbitalis	R	CT	0.003	0.027	-0.017	0.006	-0.029	-0.006
precuneus	R	CT	0.004	0.029	-0.016	0.006	-0.027	-0.005
lingual	R	CT	0.004	0.029	-0.017	0.006	-0.028	-0.005
precuneus	L	CT	0.004	0.030	-0.016	0.006	-0.027	-0.005
cuneus	R	CT	0.005	0.032	-0.016	0.006	-0.028	-0.005
insula	R	CT	0.007	0.042	-0.016	0.006	-0.027	-0.004
pars opercularis	R	CT	0.008	0.045	-0.015	0.006	-0.026	-0.004
lateral orbitofrontal	L	SA	<0.001	0.001	0.018	0.004	0.010	0.027
paracentral	R	SA	<0.001	0.004	0.019	0.005	0.010	0.029
lateral orbitofrontal	R	SA	<0.001	0.005	0.017	0.005	0.008	0.026
superior frontal	R	SA	0.002	0.027	0.013	0.004	0.005	0.021
insula	R	SA	0.002	0.027	0.014	0.005	0.005	0.023
posterior cingulate	L	SA	0.003	0.027	0.015	0.005	0.005	0.024
total	R	SA	0.003	0.027	0.014	0.005	0.005	0.024
total	L	SA	0.003	0.027	0.014	0.005	0.005	0.024
precuneus	R	SA	0.003	0.028	0.013	0.004	0.004	0.022
parahippocampal	L	SA	0.004	0.029	0.015	0.005	0.005	0.025
posterior cingulate	R	SA	0.005	0.032	0.014	0.005	0.004	0.023
paracentral	L	SA	0.005	0.032	0.014	0.005	0.004	0.023
superior frontal	L	SA	0.005	0.032	0.011	0.004	0.003	0.019
middle temporal	L	SA	0.009	0.048	0.011	0.004	0.003	0.020

Note. Significant associations (p_{FDR}<0.05) between the SCZ-GRS computed based on effect sizes taken from a GWAS of SCZ (10) and IDPs. 95% CIs were given. *Abbreviations.* CI, confidence interval; CT, cortical thickness; FDR, false-discovery rate; GRS, genetic risk score; IDP, image-derived phenotype; L, left; PCC, posterior cingulate cortex; PGC, Psychiatric Genomics Consortium; R, right; SA, surface area; SCZ, schizophrenia; SE, standard error; Vol., volume.

Table S7

Associations between genetic risk scores and outcomes related to mental health in the UK Biobank, as calculated using the extended set of covariates

Outcome related to mental health	PleioPsych-GRS				SCZ-GRS			
	p_{FDR}-value	OR	CI_{lower}	CI_{upper}	p_{FDR}-value	OR	CI_{lower}	CI_{upper}
mood swings	**8.70×10^{-03}**	1.039	1.014	1.065	8.26×10^{-02}	1.025	1.000	1.051
miserableness	**1.20×10^{-03}**	1.049	1.023	1.076	4.22×10^{-01}	1.012	0.987	1.037
irritability	**8.69×10^{-06}**	1.074	1.045	1.103	8.75×10^{-01}	0.998	0.971	1.025
sensitivity/hurt feelings	**4.26×10^{-02}**	1.028	1.004	1.053	**1.59×10^{-02}**	1.035	1.010	1.060
fed-up feelings	**4.24×10^{-04}**	1.056	1.029	1.083	4.73×10^{-01}	1.010	0.985	1.036
nervous feelings	**1.59×10^{-02}**	1.045	1.013	1.077	2.35×10^{-01}	1.021	0.991	1.053
worrier/anxious feelings	**6.56×10^{-03}**	1.039	1.015	1.064	**3.23×10^{-03}**	1.043	1.018	1.068
tense feelings/ highly strung	**6.95×10^{-04}**	1.076	1.038	1.116	**3.27×10^{-02}**	1.047	1.009	1.086
worry too long after embarrassment	1.67×10^{-01}	1.019	0.995	1.044	4.32×10^{-01}	1.011	0.987	1.035
suffer from nerves	2.35×10^{-01}	1.022	0.989	1.057	7.12×10^{-01}	0.993	0.961	1.027
loneliness	8.56×10^{-02}	1.034	0.999	1.070	8.26×10^{-02}	1.035	1.000	1.071
guilty feelings	8.26×10^{-02}	1.027	1.000	1.055	**2.06×10^{-02}**	1.037	1.009	1.065

Note. Results of testing for associations between the GRSs and outcomes related to mental health using an extended set of covariates. Bold font indicates significant p_{FDR}-values. 95% CIs are presented. *Abbreviations*. CI, confidence interval; GRS, genetic risk score; FDR, false discovery rate; OR, odds ratio; SCZ, schizophrenia.

Table S8

Associations between genetic risk scores and outcomes related to mental health in the UK Biobank excluding samples with registered ICD-10 diagnosis and self-report of MD

Outcome related to mental health	PleioPsych-GRS				SCZ-GRS			
	p_{FDR}-value	OR	CI_{lower}	CI_{upper}	p_{FDR}-value	OR	CI_{lower}	CI_{upper}
mood swings	**0.029**	1.038	1.008	1.069	**0.029**	1.040	1.009	1.071
miserableness	**0.023**	1.043	1.012	1.075	**0.029**	1.038	1.008	1.070
irritability	**0.001**	1.069	1.035	1.105	0.916	0.998	0.966	1.031
sensitivity/hurt feelings	0.344	1.015	0.987	1.044	**0.023**	1.039	1.011	1.069
fed-up feelings	**0.029**	1.039	1.008	1.072	0.114	1.029	0.998	1.061
nervous feelings	0.240	1.026	0.988	1.066	0.104	1.038	0.999	1.078
worrier/anxious feelings	0.161	1.023	0.995	1.052	**0.001**	1.056	1.028	1.086
tense feelings/ highly strung	**0.023**	1.068	1.019	1.119	**0.029**	1.061	1.012	1.112
worry too long after embarrassment	0.344	1.015	0.987	1.044	0.344	1.015	0.987	1.044
suffer from nerves	0.916	0.997	0.956	1.040	0.766	1.008	0.967	1.052
loneliness	0.240	1.031	0.987	1.076	**0.023**	1.062	1.017	1.108
guilty feelings	0.240	1.023	0.990	1.057	**0.021**	1.052	1.018	1.086

Note. Results of testing for associations between the GRSs and outcomes related to mental health excluding samples with registered ICD-10 diagnosis and self-report of depression. Bold font indicates significant p_{FDR}-values. 95% CIs are presented. *Abbreviations*. CI, confidence interval; GRS, genetic risk score; FDR, false discovery rate; MD, major depression; OR, odds ratio; SCZ, schizophrenia.

Table S9

Associations between the SCZ-GRS based on effect sizes of a GWAS of SCZ and outcomes related to mental health in the UK Biobank

Outcome related to mental health	SCZ-GRS			
	p_{FDR}-value	OR	CI_{lower}	CI_{upper}
mood swings	0.065	1.027	1.001	1.052
miserableness	0.456	1.010	0.985	1.036
irritability	0.831	0.997	0.970	1.025
sensitivity/hurt feelings	**0.017**	1.034	1.010	1.059
fed-up feelings	0.456	1.011	0.985	1.037
nervous feelings	0.256	1.020	0.989	1.052
worrier/anxious feelings	**0.004**	1.042	1.017	1.067
tense feelings/ highly strung	**0.034**	1.046	1.009	1.085
worry too long after embarrassment	0.450	1.011	0.987	1.036
suffer from nerves	0.624	0.991	0.959	1.025
loneliness	0.062	1.038	1.003	1.075
guilty feelings	**0.022**	1.037	1.009	1.065

Note. Results of testing for associations between the GRS-SCZ based on effect sizes of a GWAS of SCZ and outcomes related to mental health. Bold font indicates significant p_{FDR}-values. 95% CIs are presented. *Abbreviations*. CI, confidence interval; GRS, genetic risk score; FDR, false discovery rate; OR, odds ratio; SCZ, schizophrenia.

Supplementary Figures

Figure S1

Effect sizes of the predominantly SCZ-associated SNPs from the second cross-disorder GWAS and the most recent GWAS of SCZ by the PGC

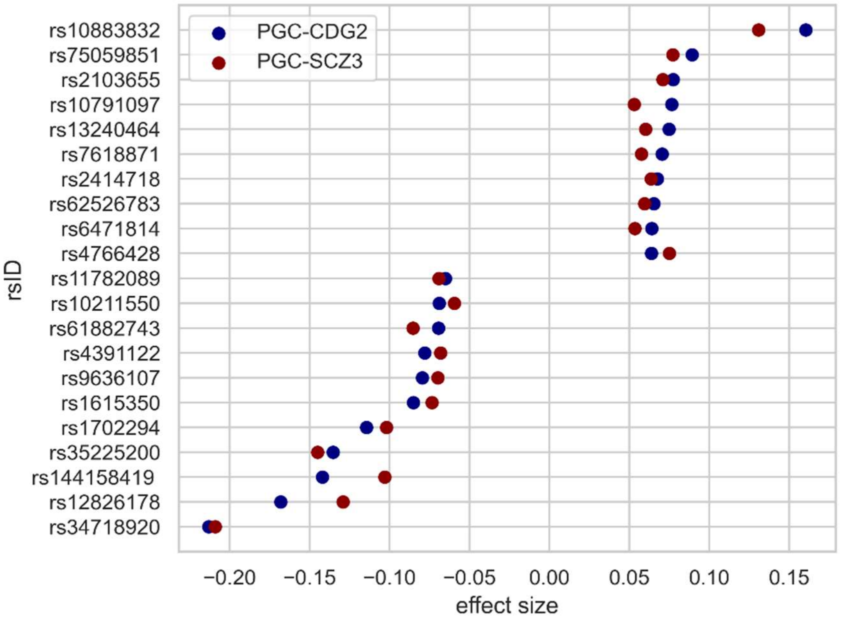

Note. Effect sizes were extracted from the summary statistics of the second cross-disorder GWAS (PGC-CDG2) (6) and the most recent GWAS of SCZ by the PGC (PGC-SCZ3) (10). Notably, rs10211550, rs11782089, and rs144158419 were not covered in the GWAS summary statistics of the PGC-SCZ3 and thus, the effect sizes of the proxy SNPs rs67657812, rs7839435, and rs72934586 were shown, respectively. As stated in Materials and Methods, the predominantly SCZ-associated SNP rs2801578 was excluded due to non-inferable allele ambiguity, while rs13217619 was replaced by rs34718920 as it was not covered in the GWAS summary statistics of the PGC-CDG2 (6). *Abbreviations*. PGC, Psychiatric Genomics Consortium; SCZ, schizophrenia.

References

1. Manichaikul A, Mychaleckyj JC, Rich SS, Daly K, Sale M, Chen WM. Robust relationship inference in genome-wide association studies. *Bioinformatics*. 2010; **26**:2867–73.
2. Primus SA, Hoffstaedter F, Raimondo F, Eickhoff SB, Winkelmann J, Oexle K, et al. Beyond volume: Unraveling the genetics of human brain geometry. *medRxiv*. 2024;
3. Dale AM, Fischl B, Sereno MI. Cortical Surface-Based Analysis: I. Segmentation and Surface Reconstruction. *Neuroimage*. 1999; **9**:179–94.
4. Fischl B, Salat DH, Busa E, Albert M, Dieterich M, Haselgrove C, et al. Whole brain segmentation: Automated labeling of neuroanatomical structures in the human brain. *Neuron*. 2002; **33**:341–55.
5. Desikan RS, Ségonne F, Fischl B, Quinn BT, Dickerson BC, Blacker D, et al. An automated labeling system for subdividing the human cerebral cortex on MRI scans into gyral based regions of interest. *Neuroimage*. 2006; **31**:968–80.
6. Lee PH, Anttila V, Won H, Feng YCA, Rosenthal J, Zhu Z, et al. Genomic relationships, novel loci, and pleiotropic mechanisms across eight psychiatric disorders. *Cell*. 2019; **179**:1469–82.
7. Wray NR, Ripke S, Mattheisen M, Trzaskowski M, Byrne EM, Abdellaoui A, et al. Genome-wide association analyses identify 44 risk variants and refine the genetic architecture of major depression. *Nat Genet*. 2018; **50**:668–81.
8. Lin LIK. A Concordance Correlation Coefficient to Evaluate Reproducibility. *Biometrics*. 1989; **45**:255.
9. Bhattacharjee S, Rajaraman P, Jacobs KB, Wheeler WA, Melin BS, Hartge P, et al. A subset-based approach improves power and interpretation for the combined analysis of genetic association studies of heterogeneous traits. *Am J Hum Genet*. 2012; **90**:821–35.
10. Trubetskoy V, Pardiñas AF, Qi T, Panagiotaropoulou G, Awasthi S, Bigdeli TB, et al.

Mapping genomic loci implicates genes and synaptic biology in schizophrenia. *Nature*. 2022; **604**:502–8.

11. Machiela MJ, Chanock SJ. LDlink: A web-based application for exploring population-specific haplotype structure and linking correlated alleles of possible functional variants. *Bioinformatics*. 2015; **31**:3555–7.

12. Ghoussaini M, Mountjoy E, Carmona M, Peat G, Schmidt EM, Hercules A, et al. Open Targets Genetics: systematic identification of trait-associated genes using large-scale genetics and functional genomics. *Nucleic Acids Res*. 2021; **49**:D1311–20.

13. Mountjoy E, Schmidt EM, Carmona M, Schwartzentruber J, Peat G, Miranda A, et al. An open approach to systematically prioritize causal variants and genes at all published human GWAS trait-associated loci. *Nat Genet*. 2021; **53**:1527–33.

14. Boyle AP, Hong EL, Hariharan M, Cheng Y, Schaub MA, Kasowski M, et al. Annotation of functional variation in personal genomes using RegulomeDB. *Genome Res*. 2012; **22**:1790–7.

15. Grasby KL, Jahanshad N, Painter JN, Colodro-Conde L, Bralten J, Hibar DP, et al. The genetic architecture of the human cerebral cortex. *Science*. 2020; **367**.

16. Satizabal CL, Adams HHH, Hibar DP, White CC, Knol MJ, Stein JL, et al. Genetic architecture of subcortical brain structures in 38,851 individuals. *Nat Genet*. 2019; **51**:1624–36.

17. Hibar DP, Adams HHH, Jahanshad N, Chauhan G, Stein JL, Hofer E, et al. Novel genetic loci associated with hippocampal volume. *Nat Commun*. 2017; **8**:13624.
